# An Acceptance‐Based Guided Self‐Help Program for Weight Loss Maintenance in Adults Who Have Previously Completed a Behavioral Weight Loss Program: The SWiM Feasibility Study

**DOI:** 10.1002/osp4.70048

**Published:** 2025-03-22

**Authors:** Rebecca A. Jones, Julia Mueller, Rebecca Richards, Jennifer Woolston, Marie Stubbings, Fiona Whittle, Andrew J. Hill, Carly A. Hughes, Robbie Duschinsky, Stephen J. Sharp, Michelle Chester, Carlotta Schwertel, Struan Tait, Patricia Eustachio Colombo, Laura Kudlek, Clare E. Boothby, Jennifer Bostock, Penny Breeze, Alan Brennan, Francesco Fusco, Emma R. Lawlor, Stephen Morris, Simon J. Griffin, Amy L. Ahern

**Affiliations:** ^1^ MRC Epidemiology Unit University of Cambridge Cambridge UK; ^2^ Division of Psychological and Social Medicine School of Medicine University of Leeds Leeds UK; ^3^ Primary Care Unit Department of Public Health and Primary Care University of Cambridge Cambridge UK; ^4^ Public Involvement Lead, Quality Safety Outcomes Policy Research Unit University of Kent Oxford and Leeds Kent UK; ^5^ Sheffield Centre for Health and Related Research (SCHARR), School of Medicine and Population Health University of Sheffield Sheffield UK; ^6^ Broadstreet Health Economics & Outcomes Research Vancouver Canada

**Keywords:** acceptance and commitment therapy, digital, interventions, obesity, overweight, weight loss maintenance, weight management

## Abstract

**Background:**

Most weight lost during weight‐loss programmes is eventually regained. Interventions based on Acceptance and Commitment Therapy (ACT) demonstrate good evidence for long‐term weight loss, but are often costly and difficult to scale up. Guided self‐help programmes delivered using technology and non‐specialist coaches could increase scalability, but it is unclear whether delivering ACT‐based interventions in this way is feasible and acceptable.

**Methods:**

In this feasibility study, 61 people who recently completed a behavioral weight management intervention (BWMI) for weight management were randomly allocated to SWiM (“Supporting Weight Management”: 4‐month digital guided self‐help ACT‐based intervention for weight loss maintenance) or a standard care group (leaflet about maintaining weight loss) using a 2:1 allocation ratio. At baseline and 6 months, participants completed measures of weight, mental health, eating behavior, and other psychosocial variables. Participants completed an intervention evaluation questionnaire. At 3 and 6 months, qualitative interviews were conducted with participants from both trial arms and SWiM coaches. The analysis integrated statistics and thematic analysis, informed by the Medical Research Council (MRC) framework for process evaluations. Since this was a feasibility study, analyses focused on process outcomes instead of interpreting statistical significance.

**Results:**

Eighty‐eight percent (36/41) of participants allocated to SWiM completed at least the first session and 22 (54%) completed all sessions. At 6 months, mean weight change was −2.2 (+/−6.4 SD) kg in SWiM participants and +2.2 (+/−6.6) kg in standard care participants. Descriptively, eating behavior and mental health scores improved in SWiM participants but not in standard care participants. In interviews, SWiM participants noted that they reinforced their existing knowledge while acquiring new skills and strategies, which were felt to contribute to positive behavioral changes.

**Conclusion:**

The SWiM intervention is practical and well‐received, and shows promise in supporting weight loss maintenance, though evaluation in a larger trial is needed to assess effectiveness.

**Trial Registration:**

ISRCTN12685964

## Graphical abstract

Maintenance of weight loss is a major public health challenge. Interventions based on Acceptance and Commitment Therapy (ACT) have shown good evidence for long‐term weight loss maintenance, but are usually too costly and reliant on specialists to scale up. We present findings from a mixed‐methods feasibility study which evaluated the acceptability and feasibility of a new ACT‐based weight loss maintenance program (“SWiM”), which uses digital technology and trained non‐specialists to reduce costs and increase scalability.

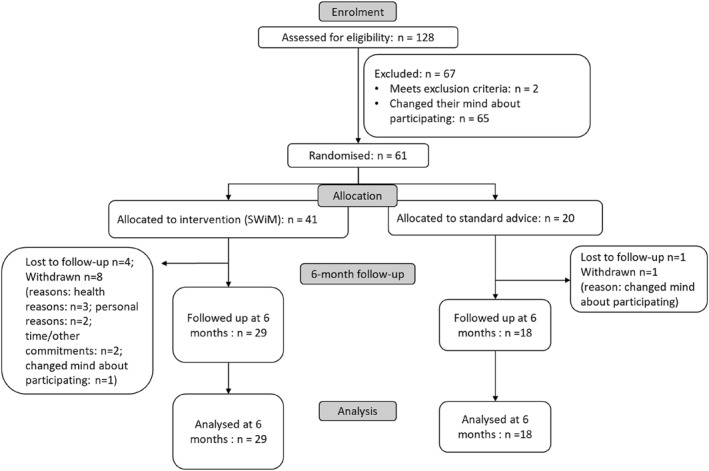

## 1 Background

Evidence suggests that behavioral weight management interventions (BWMIs) that focus on self‐regulation are cost‐effective in reducing weight [1, 2]. However, even after programmes led by specialists, most of this weight is typically regained within a few years [3]. Providing additional assistance to minimize weight regain and sustain long‐term weight loss could lead to better long‐term physical and mental health, as well as increase the cost‐effectiveness of weight management programmes [3].

Evidence suggests that interventions based on Acceptance and Commitment Therapy (ACT) principles might offer greater effectiveness for maintaining weight loss than standard BWMIs, with additional beneficial effects for mental wellbeing [4, 5]. These interventions aim to increase an individual's willingness and capacity to deal with difficult thoughts and emotions (e.g., cravings) by applying mindfulness and acceptance strategies [6]. This is theorized to facilitate the adoption and maintenance of healthy behaviors. This notion is supported by previous research from Forman and colleagues that found that a greater proportion of those receiving an ACT‐based BWMI (compared to standard BWMI) maintained 10% weight loss at 36 months, with greater quality of life also reported at 24 and 36 months [7, 8].

Several studies have indicated that ACT‐based interventions for weight management are generally well‐received [9, 10, 11, 12]. However, acceptance‐based interventions are typically administered in person by specialists like psychologists, rendering them costly and difficult to scale up and offer more widely. For example, a randomized feasibility study (*N* = 80 recruited) of an in‐person group‐based ACT intervention in people after bariatric surgery found high dropout rates from the intervention, and qualitative interviews highlighted that reasons included opportunity and travel costs associated with attending the intervention in person [13]. Interventions that can be offered widely at low cost are urgently needed. Digital, guided self‐help interventions administered by non‐specialists (e.g., trained lay coaches) could lower costs and expand accessibility. Existing evidence indicates that remotely‐delivered obesity treatments are generally feasible and acceptable, though impacts on weight are mixed [14, 15]. A pre‐post feasibility study on treating weight regain in bariatric surgery patients (*N* = 20 enrolled in the program) found a remote web‐based ACT‐based intervention to be both feasible and acceptable [12]. Other randomized trials of digital ACT‐based interventions for lifestyle modification have generally highlighted their acceptability and feasibility, with positive effects on eating behaviors and psychological flexibility, but effects on weight and physical activity are unclear [16, 17, 18, 19]. This highlights the potential of remotely‐delivered ACT‐based interventions for preventing weight regain though further research is needed.

The first‐line treatment for people with overweight or obesity is standard BWMI. The present study examined the potential of using an ACT‐based intervention to support weight loss maintenance following a standard BWMI for weight loss. ACT‐based interventions could help reinforce strategies learned during standard BWMI and help address key challenges to adhering to such strategies long‐term, such as emotion regulation [20].

The current study aimed to evaluate the feasibility and acceptability of a digital self‐help intervention which is supported by trained lay coaches and draws on ACT‐based principles to help people maintain weight loss after a standard BWMI [21].

## 2 Methods

A comprehensive description of the study methods is available in the published protocol [22].

### 2.1 Design

This study is a pragmatic, single‐blind, two‐arm, randomized feasibility trial with an embedded interview study.

### 2.2 Eligibility

Adults (18+ years) who had, in the last 3 months, attended a BWMI (minimum duration 12 weeks), were able to provide informed consent, were able to understand study materials written in English, could access the online platform, and had access to scales for self‐weighing were recruited. Individuals were excluded based on the following criteria: using insulin, having undergone or planning to undergo bariatric surgery, currently pregnant or planning a pregnancy, and ongoing diagnosis of an eating disorder.

### 2.3 Recruitment

Participants were recruited through the National Health Service (NHS) England, as well as commercial and local authority weight management services. Eligible, willing adults were sent a secure webform to confirm eligibility and provide informed consent.

### 2.4 Interventions

#### 2.4.1 Supporting Weight Management (SWiM) Intervention

The intervention, “SWiM”, was an ACT‐based, guided self‐help intervention for weight loss maintenance. Participants received access to a web‐based platform featuring 14 sequential modules that included psychoeducational material, reflective exercises, and behavioral experiments. Participants were also provided with exercises to complete throughout the week (“SWiM Practices”). Exercises that related to core skills were stored on a designated webpage for easy access (“SWiM Aids”). Participants received four telephone calls from a trained, lay “SWiM coach” after modules one, three, eight and 14. Participants could request up to three additional calls. Details about the intervention content are available elsewhere [21, 22].

#### 2.4.2 Standard Care

Participants in the standard care control group received a leaflet about maintaining weight loss encouraging them to create their own weight‐loss maintenance plan.

### 2.5 Randomization and Blinding

Participants were randomly assigned to either the SWiM group or the standard care group in a 2:1 ratio, in blocks stratified by type 2 diabetes status and sex. Participants were blinded to their allocated intervention until they had enrolled and were assigned to a group. After group assignment, it was not feasible to blind participants due to the nature of the interventions. Due to the uneven group sizes, it was not possible to blind the data analysist.

### 2.6 Outcomes and Measures

Study feasibility was examined by study uptake, recruitment rate, and retention. Intervention engagement and acceptability were examined by key engagement metrics (number of sessions completed, average time spent on each session, number and duration of coach calls) and participants' perceptions of the intervention's usefulness, helpfulness and enjoyability. The feasibility and acceptability of the study and intervention were assessed using the measures detailed in Table 1.

**TABLE 1:** Feasibility and acceptability measures.

Outcomes	Measure	Source
Uptake of the intervention	Number and proportion of participants who took up the intervention out of those who consented. Uptake of the intervention was defined as completing at least the first SWiM session [1].	Study management records
Uptake of the study	Number and proportion of participants who consented out of those who were eligible.	Study management records
Recruitment rate	Number of recruited participants per month	Study management records
Retention	Number of participants who withdrew, with reasons, and the number of participants with missing data at follow‐up, separately for each study group.	Study management records
Intervention engagement	Average number of sessions completed Average time spent per session Duration of use of the SWiM platform (time between first and last login to the platform)	Website analytic data
Intervention engagement and acceptability	Average number of coach calls per participant Average duration of coach calls Summary of call content (open‐text response) Open‐text responses (e.g., explanations from coaches regarding why they were unable to deliver calls as intended; any additional notes)	Coach call report form completed by coaches

In addition, semi‐structured telephone interviews were conducted with both SWiM coaches and 18 participants from the intervention and 10 participants from the control group at intervention mid‐point (3 months post‐baseline). At post‐intervention (6 months post‐baseline), 15 out of 18 intervention participants and both SWiM coaches (*n* = 2) participated in a further qualitative interview. All intervention participants who withdrew from the study were invited to participate in a qualitative interview (*n* = 8 invited, *n* = 3 interviews conducted). Purposive sampling was conducted for variation in age, sex, ethnicity, and occupation among intervention and standard care participants interviewed (Supporting Information S1: Table S1).

Researchers (RR, LK, JM) trained in qualitative methods and with expertise in weight management research conducted the telephone interviews. RR developed, piloted and revised the interview schedules with support from RAJ, RD, and the wider research team. Participants provided informed consent to participate in the qualitative interviews. Interviews were audio‐recorded with participants' permission and transcribed verbatim by an experienced external transcription agency.

To guide the design of a future effectiveness trial, changes in the proposed primary and secondary outcomes were assessed using self‐report questionnaires. The proposed primary outcome for the future effectiveness trial was change in self‐reported weight (kg); participants were asked to measure their height and weight using a standardized protocol and report this on the same day. Secondary outcomes are shown in Table 2.

**TABLE 2:** Secondary outcome measures.

Outcome	Measure	Score range	Higher scores indicate
Depressive symptoms	Patient health questionnaire (PHQ‐8 [23])	0–24	Higher symptom severity
Anxiety symptoms	Generalized anxiety disorder 7‐item scale (GAD‐7 [24])	0–21	Higher symptom severity
Stress	Perceived stress scale (PSS‐4 [25, 26])	0–16	Higher stress levels
Eating behavior (cognitive restraint of food intake, uncontrolled eating, emotional eating)	Three‐factor eating questionnaire (TFEQ‐R21) [27]	0–100 on each subscale	Higher levels of each eating behavior
Experiential avoidance/psychological flexibility	Acceptance and action questionnaire weight related‐revised (AAQW‐R [28])	10–70	More experiential avoidance and less psychological flexibility
Capability wellbeing	ICEpop CAPability measure for adults (ICECAP‐A [29])	0–1	Higher wellbeing
Habit strength (automaticity) for eating high‐calorie snack foods in response to emotions	Bespoke questionnaire, adapted from the self‐report behavioral automaticity index [30], see questionnaire in supplementary materials	10–70	Stronger automaticity

### 2.7 Data Analysis

#### 2.7.1 Quantitative Analysis

For continuous variables, means and standard deviations (SDs) are presented. For categorical variables, the number and percentage of participants per category are provided. To guide an estimate for an appropriate sample size for a future cost‐effectiveness trial of the SWiM intervention, linear regression was conducted to estimate the difference between the two groups in weight change (baseline to 6 months follow‐up), controlling for baseline weight and the variables used to stratify randomization (diabetes status and sex), following the intention‐to‐treat principle.

#### 2.7.2 Qualitative Analysis

NVivo 12 Pro (QSR International) was used to organize and manage the data, informed by the Medical Research Council (MRC) framework for process evaluations. RAJ led the data analysis. A sample of transcripts were second coded by ST and PEC. Reflexive thematic analysis was conducted to discern patterns of meaning across the dataset [31]. Mid‐intervention interviews were analyzed first, with the thematic framework then guiding the analysis of the post‐intervention interviews. The thematic framework from the analysis of the SWiM coach interviews was used to guide the analysis of the coach call report forms. The thematic frameworks were adapted iteratively throughout the analysis process.

In the results, quotes from interviews are identified by “SWiM” (intervention participants), “SC” (standard care group participants), WD (withdrawn intervention participants), or “SWiM Coach.”

#### 2.7.3 Integration of Quantitative and Qualitative Findings

Qualitative and quantitative results from different data sources were integrated using a joint display table to generate meta‐themes [32]. To support this process, the following were examined: (1) the level of agreement, (2) how findings from one source complemented findings from the other source, (3) instances where findings from the two sources were in dissonance, (4) areas of “silence” (i.e., themes emerging from one source but not the other).

### 2.8 Ethical Approval

Cambridge South Research Ethics Committee granted ethical approval (ref: 21/EE/0024).

## 3 Results

### 3.1 Trial Uptake and Withdrawal

**FIGURE 1:**
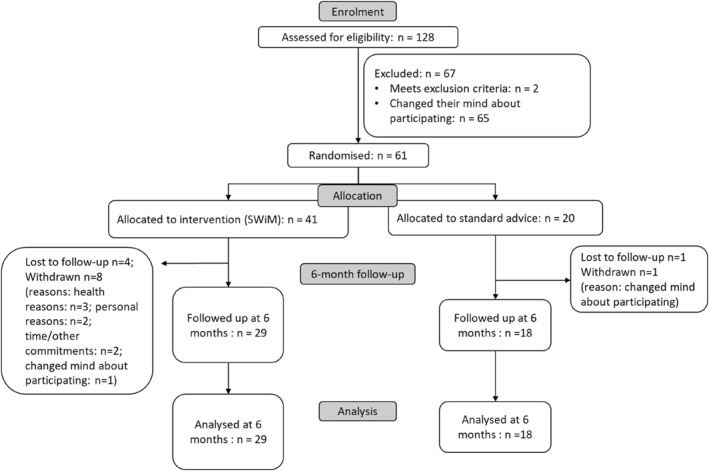
Consort flowchart.

Recruitment continued until the target sample (*N* = 60) [22] was reached. Between 19th May and 9^th^ November 2021, 128 people were assessed for eligibility. Out of those who were eligible (*n* = 126), 61 (48%) were recruited (SWiM: *n* = 41, Control: *n* = 20, Figure 1). The average recruitment rate was 10.2 participants per month. Although three sites were actively recruiting, 98% (126/128) of those who expressed interest and 98% (60/61) of the recruited sample were from one site. High levels of engagement from this service provider, and direct recommendation of the intervention to service users, were identified as key facilitators of recruitment. Reasons for non‐participation following expression of interest included unwillingness to be randomized and feeling overwhelmed at the thought of participating in a “*psychological*” study (Recruitment notes).

Table 3 shows participant characteristics. One participant withdrew from the standard care group. In the SWiM group, 8 participants withdrew and 4 were lost to follow‐up. At the time of withdrawal, the reasons given were difficulty balancing their time and commitments, health problems, and dissatisfaction with the intervention. Similarly, in the 3 withdrawal interviews, reasons for withdrawing from the study included experiencing ill health (e.g., hospitalizations), struggling to manage multiple commitments (e.g., work, family), and dissatisfaction with the intervention.

**TABLE 3:** Sample baseline characteristics. Numbers shown are *n* (%) unless stated otherwise.

		Standard care, *n* = 20	SWiM, *n* = 41	Total sample, *N* = 61
Age (years)	*M*, SD	47.3 (12.9)	48.3 (14.8)	48.0 (14.1)
Sex	Male	2 (10%)	8 (20%)	10 (16%)
	Female	18 (90%)	33 (81%)	51 (84%)
Ethnicity	White	19 (95%)	39 (95%)	58 (95%)
	Non‐White	0 (0%)	2 (5%)	2 (3%)
	Prefer not to say	1 (5%)	0 (0%)	1 (2%)
Marital status	Single	1 (5%)	9 (22%)	10 (16%)
	Married/Civil partnership/Co‐habiting	19 (95%)	20 (49%)	39 (64%)
	Widowed	0 (0%)	2 (5%)	2 (3%)
	Separated/Divorced	0 (0%)	9 (22%)	9 (15%)
	Prefer not to say	0 (0%)	1 (2%)	1 (2%)
Occupation	Employed (full or part‐time or self‐employed full or part‐time)	15 (75%)	23 (56%)	38 (62%)
	On a government supported training program/Full‐ or part‐time education at school college or university	0 (0%)	1 (2%)	1 (2%)
	Currently not employed	2 (10%)	9 (22%)	11 (18%)
	Retired	3 (15%)	8 (20%)	11 (18%)
Education	Below post‐secondary (up to and including A‐levels)	10 (50%)	21 (51%)	31 (51%)
	Post‐secondary	10 (50%)	19 (46%)	29 (48%)
	Prefer not to say	0 (0%)	1 (2%)	1 (2%)
Cost of living situation	Find it a strain to get by from week to week	2 (10.0%)	3 (7.3%)	5 (8.2%)
	Have to be careful about money	10 (50%)	19 (46%)	29 (48%)
	Able to manage without much difficulty	3 (15%)	12 (29%)	15 (25%)
	Quite comfortably off	4 (20%)	5 (12%)	9 (15%)
	Prefer not to say	1 (5%)	2 (5%)	3 (5%)
Diabetes status	Diagnosed with diabetes	3 (15%)	4 (10%)	7 (12%)
	Don't know	0 (0%)	1 (2%)	1 (2%)
BMI	*M*, SD	39.1 (8.3)	38.8 (9.0)	38.9 (8.7)
BMI group	≥ 30 kg/m^2^	19 (95%)	36 (88%)	55 (90%)

Abbreviations: *M*, mean; *n*, number of participants; SD, standard deviation.

### 3.2 Intervention Engagement

On average, participants used SWiM for 153 days, though variation was large (SD = 114). Most (27/41, 66%) used the web platform for the intended number of days (119 days) or longer. Two participants logged in but did not use the program again, and two participants did not log in at any point. Of those randomized to SWiM, 88% (36/41) completed the first session. Thirty participants (73%) completed ≥ 3 sessions, 28 (68%) completed ≥ 8 sessions, and 22 (54%) completed all 14 sessions. On average, participants completed 9.7 sessions (SD = 5.7). Following removal of outliers (instances with session time ≥ 3 h, where the computer was likely left unattended; excluded 13 out of 615 recorded session times), the average time per session was 16.1 min (SD = 23.3).

Thirty‐eight participants (93%) had contact with a SWiM coach at least once. Twenty‐four (59%) received all four scheduled coach calls and 21 received at least one optional call. Three participants received more than 3 optional calls (4 calls: *n* = 2, 7 calls: *n* = 1), although the SWiM intervention protocol defined a maximum of 3 additional calls per participant. Following this up with the coaches, it was reported that some of these were engagement calls (i.e., calls to encourage participants to re‐engage with intervention content), which became longer calls. On average, participants had 4.9 telephone calls with the coach. The average duration per call was 20.4 (SD = 9.4) minutes, with a mean total telephone contact time of 106.0 min (SD = 65.9) per participant.

In interviews, SWiM coaches described that it was “*very rare that [the coach] makes a phone call and [the participant is] at the point they need to be at*” (SWiM Coach—3 months), suggesting that participants were not completing sessions at the anticipated pace. According to coaches, barriers to engagement included experiencing health problems, work/study stresses, family pressures, travel, mental health, receiving bad news/dealing with personal issues, and dissatisfaction with the intervention.

Participant is happy with the contents of the program, however they have had a very hectic time due to work, family circumstances and health reasons, so they did not have too much time to engage. It was quite evident that they have a lot of things to juggle and sometimes not much mental space to be as reflective as they would like.

(Coach Report)

To support participant engagement, the SWiM coaches completed engagement calls when participants had “*fallen behind and [had] not been completing sessions*” to “*motivate them to complete the sessions*” (SWiM Coach—3 months). Coaches described the participants as being “*quite grateful to receive the call just as a nudge or as a reminder*” as it provided an “*opportunity to raise any issues or concerns that they may be having with the intervention*” (SWiM Coach—3 months). The coaches emphasized the importance of being empathetic and encouraging by trying to “*ask some questions around how [the participants] could perhaps manage to integrate [SWiM] in their schedule*” (SWiM Coach—3 months). Optional calls were used to reduce the gap between the scheduled calls at sessions 3 and 8. Coaches perceived this to be too long of a gap between contacts.

The coaches suggested that engagement could be improved by allowing participants to define the pace of the intervention themselves and letting participants choose which intervention content to engage with, depending on perceived relevance. Similarly, participants described feeling restricted by the requirement to finish one session before accessing the next, describing the intervention as too “*regimented*” (WD—3 months).

### 3.3 Effects of the Intervention

At 6 months, SWiM participants lost an average of 2.2 kg (+/−6.4), while standard care participants gained 2.2 kg (+/−6.6) (Supporting Information S1: Figure S2, Table 4). The adjusted difference between the study groups was −3.8 kg (95% CI: −7.7 to 0.1 kg, *p* = 0.06). The distribution of weight change across the two groups is shown in Supporting Information S1: Figures S1 and S2. Descriptively, there were observed reductions in perceived stress, depressive symptoms, and anxiety symptoms for SWiM participants over the 6 months, while the standard care group showed increases in these variables. Wellbeing increased in the intervention group and decreased slightly in the standard care group. Emotional and uncontrolled eating and experiential avoidance decreased in the intervention group, and increased slightly in the standard care group (Table 4, Supporting Information S1: Figure S3). The self‐reported habit index score decreased in both groups; the intervention group had a larger decrease in this score than the standard care group (Table 4). Mean cognitive restraint of food intake appeared to increase slightly in both groups from baseline to follow‐up (Supporting Information S1: Figure S4). As this was a small‐scale feasibility trial, no assessment of whether these differences between SWiM and standard care groups were statistically significant was conducted.

**TABLE 4:** Outcomes at baseline and 6‐month follow‐up, and change in outcomes from baseline to follow‐up.

Outcomes		Baseline		6 months		*n*	Change from baseline to 6 months *M* (SD)
		*n*	Mean, SD	*n*	Mean, SD		
Weight	SWiM	41	107.78 (24.51)	29	100.88 (22.20)	29	−2.15 (6.43)
	SC	20	106.11 (27.11)	18	110.51 (29.64)	18	2.17 (6.60)
PHQ	SWiM	41	7.22 (5.50)	30	6.00 (6.01)	30	−1.30 (3.88)
	SC	20	10.30 (5.74)	19	10.89 (7.32)	19	0.95 (5.90)
GAD	SWiM	41	6.41 (5.37)	30	4.90 (5.17)	30	−1.73 (3.64)
	SC	20	7.75 (6.23)	19	8.58 (5.95)	19	1.42 (5.68)
PSS	SWiM	41	6.22 (3.24)	30	5.70 (2.84)	30	−0.33 (2.75)
	SC	20	7.80 (3.17)	19	8.26 (3.02)	19	0.47 (2.76)
AAQWR	SWiM	41	45.10 (14.95)	30	33.77 (14.57)	30	−11.87 (9.91)
	SC	20	47.50 (9.06)	19	47.63 (12.76)	19	−0.37 (9.80)
TFEQ—RESTRAINT	SWiM	41	55.56 (18.43)	29	57.67 (16.83)	29	−0.20 (16.50)
	SC	20	44.71 (19.20)	19	45.32 (17.30)	19	2.05 (21.12)
TFEQ—UNCONTROLLED EATING	SWiM	41	48.78 (25.07)	29	32.18 (21.36)	29	−13.42 (14.46)
	SC	20	51.86 (25.56)	19	54.58 (18.74)	19	0.19 (19.66)
TFEQ—EMOTIONAL EATING^a^	SWiM	41	59.21 (20.20)	29	48.47 (16.40)	29	−11.11 (16.48)
	SC	20	61.67 (16.99)	19	63.45 (16.28)	19	−0.58 (13.46)
ICECAP	SWiM	41	0.78 (0.17)	29	0.84 (0.13)	29	0.04 (0.10)
	SC	20	0.78 (0.11)	19	0.76 (0.14)	19	−0.02 (0.10)

Abbreviations: *M*, mean; *n*, number of participants; SC, standard care; SD, standard deviation; SWiM, Supporting Weight Management intervention.

^a^ Although mean emotional eating in the standard care group increased from baseline to follow‐up, mean change shows a small reduction in emotional eating. This apparent inconsistency is because mean emotional eating at baseline includes all 20 participants in the standard care group, whereas mean change includes only 19 participants.

In interviews, SWiM participants described experiencing a range of positive changes that they attributed to the intervention, including being more active, eating a healthier diet, having more energy and an improved mood, reduced joint pain, and reduced/cessation of medications.

I think I’ve become calmer as a result of [SWiM] and I think one of those is that [SWiM metaphor about] letting go of the rope and unplugging, pulling the plug out of the sink, letting the water drain away. I let go far more now than I ever did so I’m not so stressed. I have stopped taking one of my medications as well… I don’t think I would have been able to drop that medication without the SWiM programme.

(SWiM15—3 months: Female, 50–59y)

Participants shared that the knowledge and skills from both their prior program and the SWiM intervention helped them to create new habits. They described that SWiM taught new acceptance‐based skills and strategies , which in turn, supported behavioral changes, such as developing acceptance of things beyond their control, considering decisions as a “choice point,” using mindful breathing, urge surfing, and developing willingness to experience difficult emotions (e.g., cravings, wet weather when exercising). Additional skills and strategies noted as useful included emotional response planning, setting realistic goals, and developing assertiveness with their family and friends.

### 3.4 Perceptions of the SWiM Intervention

Thirty‐three participants completed the post‐intervention questionnaire. Participants rated the intervention as useful (6.0 +/1.5), easy to use (mean: 5.9 +/−1.3), and enjoyable (5.7 +/1.4). The majority rated the individual components of SWiM as useful/very useful (SWiM sessions: 27/33, 81.8%, SWiM Practices: 25/33, 75.7%, SWiM Aids: 24/33, 72.7%; Weight tracker: 22/33, 66.6%).

In interviews, participants described particular content as useful such as habits, coping strategies, emotional eating, stigma, goal‐setting, planning, self‐acceptance, and managing lapses. The SWiM practices reinforced the session content, with the metaphors and questions aiding understanding of the content. Conversely, some perceived the content as repetitive and patronizing, and as having too many questions and sections that required their input. Some participants suggested that the content could be improved by tailoring it to the individual participant (e.g., personalized dietary advice).

Perceptions of the imagery within the intervention content varied. Whilst some found the images “*really powerful*” (SWiM15—3m: Female, 50–59y) and helpful to understand the metaphors, others found the images to be childish, non‐relatable, and too cartoony.

I found [the images] a little childish… I like some of the ideas behind it but I did feel as if it was talking down to me at times… it was a bit condescending

(SWiM8—3 months: Female, 50–59y)

Most participants rated the coach contact as useful/very useful (Coach calls: 28/33, 84.8%, Coach emails: 23/33, 69.7%). In interviews, participants perceived the SWiM coaches to be an important component of the intervention. They described the coaches as providing accountability, helping them to understand content and work through problems, and providing motivation and encouragement. For example, coaches were described as providing advice, reviewing goals and progress, checking in on participants' mental health, acknowledging achievements and celebrating successes, and rephrasing content to aid understanding.

### 3.5 Intervention Delivery Methods

The intervention content was primarily delivered via the SWiM web platform. This was used largely at home (27/29, 93%); some (4/29, 14%) also indicated using it at work. Most participants (18/29, 62%) did not access the web platform during a set time. Most participants accessed SWiM via a laptop (13/29, 45%) and/or via a smartphone (15/29, 52%). In interviews, participants described the platform as easy to use and highlighted the logical layout, clear homepage, the use of bullet points and relevant imagery, and the use of plain English as features that improved user experience.

Most questionnaire respondents (24/29, 83%) did not experience any technical issues with SWiM. However, in interviews, participants described considerable technical problems and frustrations primarily related to using the platform on a mobile device rather than a laptop or computer. Participants suggested that the platform could be improved by developing the intervention into an app.

I wanted an app so you could link on your app or get daily reminders of hints and tips or images or whatever… I thought if it was on an app you could quickly open it and add to it, but it’s a bit more cumbersome being in the website.

(SWiM15—3 months: Female, 50–59y)

Remote participant support was delivered by SWiM coaches. Particular skills and attributes were identified as supporting coaches in their role, such as listening, reflecting, motivational interviewing, and having an approach which was open‐minded, highly empathetic, and encouraging. Furthermore, having a good understanding of stigma and discrimination was described as important as “*this topic can be very sensitive and very delicate*” and doing so can help to “*build trust and build a sort of rapport*” (SWiM Coach—3 months).

Coaches described finding it difficult to not fall “*in the trap of giving advice*” (Coach Report) and instead use their motivational interviewing skills. Coach training to deliver the remote support was described as very comprehensive. In terms of ongoing support, the SWiM coaches described that they regularly had supervision to assess their support needs. It was suggested that the supervision could be improved by being more structured. For example, coaches suggested reviewing participant cases and problem solving together so that their line manager could provide higher‐level knowledge of the underpinning intervention concepts (e.g., ACT).

### 3.6 Experience of the Standard Care Group

When allocated to the standard care group (leaflet), participants described feeling disappointed with their allocation, although they articulated an understanding of the need for a standard care group in a trial. Most participants could not recall the information within the leaflet they were given and did not engage with the leaflet. Those who could recall the content of the leaflet described the content to be useless, very limited, and as providing nothing new. Participants described feeling disappointed and lacking support and accountability. Some participants shared that they felt more alone and like nothing will ever work for them.

I read it when I first got it. I haven’t really gone back to it, I didn’t find it very encouraging or motivating. It wasn’t something that that was going to really help me… It was a bit sort of dry and didn’t really push me to think more, or encourage me to do anything.

(SC2—6 months: Female, 40–49y)

Some standard care participants described engaging with other weight management programmes during the study period (e.g., Slimming World and Noom). Participants shared that these programmes supported them to make dietary changes and ultimately lose weight. At 6 months, 10/19 participants in the standard care group and 12/29 in the intervention group reported accessing additional weight management resources (i.e. commercial/NHS/other weight loss group, gym/sport club membership, dietitian). It should be noted that this question did not cover digital resources such as weight management apps.

## 4 Discussion

In this mixed methods evaluation of a self‐help intervention for weight loss maintenance with guided support by non‐specialist coaches (SWiM), results suggest that the intervention was feasible to deliver, acceptable to most participants, and appeared to work as intended. Some preliminary indications identify that the intervention could support weight loss maintenance after a BWMI.

Most participants engaged well with the SWiM content and the coach calls; 88% of participants completed at least the first session and 93% had contact with a coach. This highlights high intervention uptake and is comparable to other digital weight management trials [33]. In this trial, 54% of participants completed all 14 sessions of the intervention. Completion rates were comparable to those in other studies involving guided digital, ACT‐based interventions for weight management [18, 34, 35], and higher than in a digital ACT‐based intervention without guided support [10]. A remotely delivered ACT‐based intervention to support patients after bariatric surgery found 60% of participants completed 8 out of 10 modules [12]; this is comparable to the completion rate in the current study. In a randomized controlled trial of an ACT‐based intervention aiming for weight loss in adults with obesity, findings showed 77% of participants engaged in 25 or more of the 30 scheduled group ACT sessions [8]. A higher completion rate may have been evident due to the higher experience level of the session leaders and the in‐person nature of the groups. Previous research has highlighted that including some form of human contact in web‐based interventions is likely to increase engagement and effectiveness [15, 36]. In line with this, qualitative insights suggested that the coach played an important role in engagement with SWiM. Future research could examine the level of engagement needed to achieve the intended outcomes [37].

Most participants found the SWiM intervention to be useful and enjoyable. Participants described finding the ACT‐based sessions helpful, including content on coping strategies, emotional eating, weight stigma, and self‐acceptance. This aligns with the findings of a qualitative review investigating weight management interventions in adults with severe obesity [38], which reported that participants liked aspects of the intervention that increased self‐awareness and helped them reflect on eating behaviors [38]. Similarly, “Project HELP” (a remotely delivered ACT‐based intervention for bariatric surgery) reported high acceptability ratings, with the highest ratings for strategies associated with ACT (e.g., acceptance, willingness, mindful decision‐making) [12]. Participants of the SWiM intervention also shared that coaches were an important element of the intervention; coaches were described as providing motivation, accountability, and support to better understand intervention content. Findings of a qualitative evaluation of remotely delivered weight management support (via telephone calls and text messages) align with this [39]; participants unanimously agreed that the remote support offered necessary encouragement and motivation, whilst reducing the usual barriers to in‐person support such as traveling or taking time off work [39]. Overall, SWiM content and coach contact were deemed acceptable and were well‐received by participants and providers.

Differences between groups in outcomes were consistently in the expected direction, suggesting SWiM may improve weight loss outcomes and multiple psychological and behavioral determinants of long‐term weight management. These findings are supported by an earlier randomized trial of a “light” version of SWiM (i.e., with less content and coach support) adapted to support people with weight management during the COVID‐19 pandemic. In SWiM‐COVID, statistically significant differences between groups were observed in psychological flexibility, eating behavior (uncontrolled and emotional eating), cognitive restraint, wellbeing and physical activity at 4 and/or 12‐month follow up [17, 40]. Long‐term follow‐up of an ACT‐based BWMI for obesity found significant improvement in quality of life at 24 and 36 months, compared to standard BWMI [7]. This further supports that this form of intervention shows promise for improving both psychological and physical health outcomes.

Qualitative insights also highlighted non‐weight related benefits such as improved mood and reduced pain. Two recent systematic reviews concluded that ACT‐based approaches to weight management show promise to benefit weight loss, psychological flexibility, weight stigma, and psychological wellbeing [41, 42]. This indicates the potential SWiM has to benefit both physical and mental health in the longer term, and reinforces the decision to collect mental health outcomes as part of a future trial.

Weight management interventions commonly facilitate weight loss through dietary restraint; however, there have been concerns about this increasing risk of disordered eating [43, 44]. Additionally, evidence indicates that approaches focusing on restraint alone are unlikely to effectively address the environmental and genetic obstacles to weight management [45]. Interestingly, the findings did not indicate that SWiM is likely to increase dietary restraint. Instead, the results tentatively suggest SWiM may improve psychological flexibility, emotional eating, and uncontrolled eating. In interviews, participants described how SWiM helped them engage more flexibly with their internal experiences, rather than attempting to control or avoid uncomfortable feelings, such as cravings, which supported them to enact behavioral changes. This aligns with theories of ACT for long‐term weight management, which suggest that the development of psychological flexibility through, for example, willingness, supports value‐based action in the context of challenging and uncomfortable experiences, thus supporting longer‐term weight management [6].

This feasibility study provides useful information to guide the design of a future (cost‐)effectiveness trial for SWiM. Randomization to intervention and standard care was acceptable, but standard care participants were disappointed not to receive further support. Although the use of a wait‐list control may address this disappointment, it would hamper the ability to make long‐term comparisons between groups. A future trial may consider alternative strategies to maintain standard care group satisfaction by, for example, sending more regular newsletters with trial updates. Additionally, a future trial should assess the use of any additional weight management resources (e.g., commercial weight loss apps) in both groups and consider appropriate statistical methods to account for this.

Overall, trial retention was greater or comparable to other weight management trials [2, 46, 47, 48]. Whilst the current study had a 14.8% dropout (*n* = 9/61) at post‐intervention follow‐up, the Mind Your Health trial [8] (ACT‐based vs. standard BWMI for obesity) experienced 22.7% dropout (*n* = 29/128) and Project HELP [12] (remotely delivered BWMI for weight regain after bariatric surgery) reported 45% dropout (*n* = 9/20). Despite good trial retention, the current study experienced a differential dropout between study arms. Although many participants found the intervention acceptable, the rate of drop out was higher within the intervention (*n* = 8/41, 20%) versus standard care group (*n* = 1/20, 5%). Differential dropout can result in systematic differences between intervention and standard care participants that can confound intervention effects [49]. The intervention group was asked to commit greater time and energy to engaging with the trial than the standard care group, and this may contribute toward the differential dropout rate. Intervention participants who withdrew from the trial reported struggling to engage with SWiM alongside their other demands and commitments. This aligns with the qualitative findings of the SWiM COVID trial [50]. Future trials may consider implementing strategies recommended by Robinson et al. [51] to support retention, such as using special tracking methods for lost to follow‐up participants to identify and address obstacles to participation.

This study is the first in the UK to examine the feasibility and acceptability of a digital intervention incorporating ACT to aid in maintaining weight loss after completing a BWMI. Integration of data from quantitative and qualitative sources was conducted, following MRC guidance for process evaluations [31].

The qualitative data used was from two different time points (mid‐ and post‐intervention), from multiple perspectives (intervention and standard care participants, participants that withdrew, coaches), and multiple sources (interviews, coach call report forms). Quantitative data drew on self‐reported data, objective engagement data from the web platform, and detailed intervention evaluation forms. A rigorous approach was used to integrate the findings and yield new insights [32].

Self‐reported weight was used as the trial was conducted remotely. Self‐reported weight may be under‐reported and less accurate than objectively measured weight [52], yet is similar to smart scales providing direct self‐measurements [53]. To increase the accuracy of self‐reports, clear, specific instructions were provided to participants. Additionally, since participants were randomized, it is assumed that any discrepancies in reporting are uniformly spread across the groups.

In this study, the majority of participants were White and female. Although lack of diversity is common within behavioral weight management trials [54, 55], it limits the representativeness of the sample for the broader population eligible for such services. As recruited participants had recently completed a BWMI, recruitment was restricted to the samples within these services. However, to increase sample diversity in a future trial of (cost‐)effectiveness, inclusive and intentional recruitment strategies, co‐designed with a diverse panel of people with lived experience, will be utilized.

### 4.1 Conclusion

The findings indicate that the SWiM intervention was acceptable and feasible and demonstrated the potential to improve weight loss maintenance following a BWMI. Participants reported improvements in psychological and behavioral determinants of long‐term weight management, which highlights SWiM's potential to benefit both physical and mental health in the longer term. Areas for refinement were identified (e.g., optimizing the web platform for mobiles) which can inform future intervention development and trial design.

## Author Contributions

Conceptualization: A.L.A., S.J.G., A.J.H., C.A.H., R.D.; Study design: A.L.A., S.J.G., A.J.H., C.A.H., R.D., S.J.S., R.A.J., J.M., R.R., J.W.; Intervention development: R.R., R.A.J., A.L.A., S.J.G., F.W., A.J.H., C.A.H.; Data collection: R.A.J., J.M., L.K., J.W., F.W., M.S., C.E.B.; Data analysis: R.A.J., J.M., C.S., S.T., P.E.C., R.D., S.J.S.; Initial manuscript drafting: R.A.J., J.M., A.L.A.; trial operations and management: J.W., M.S., F.W., M.C., C.S.; Intervention delivery: M.C., C.S.; Data curation and management: C.E.B.; Chaired the patient and public involvement group: J.B.; Study supervision: A.L.A., S.J.G.; Writing the manuscript and approving submitted and published versions: All.

## Conflicts of Interest

F.W., J.W., R.D., S.J.S., S.M., C.E.B., P.E.C., P.B., A.B., F.F., E.L., L.K., R.R. and J.B. declare no conflicts of interest. Since the trial analyses were completed, RAJ has started working for WW (Weight Watchers). A.J.H. has served as a consultant for Slimming World. C.A.H. has received consulting fees, honoraria, or support for attending meetings or travel from Ethicon and Novo Nordisk. J.M. is a member of the Operations Committee for the Association for the Study of Obesity (ASO) in an unpaid role and has organized educational events for ASO members with funding from Boehringer Ingelheim Ltd and Rhythm Pharmaceuticals. A.L.A. and S.J.G. are chief investigators for two publicly funded trials (MRC and NIHR) where WW provides the intervention at no cost. A.L.A. is a member of WW’s Scientific Advisory Board. S.J.G. has received honoraria from AstraZeneca and Eli Lilly for contributing to postgraduate educational meetings and serves as a Trustee for the Novo Nordisk UK Research Foundation.

## Rights Retention Statement

For the purpose of Open Access, the author has applied a Creative Commons Attribution (CC BY) license to any Author Accepted Manuscript version arising.

## Supporting information

Supporting Information S1
