# Supplementary material for: An Acceptance‐Based Guided Self‐Help Program for Weight Loss Maintenance in Adults Who Have Previously Completed a Behavioral Weight Loss Program: The SWiM Feasibility Study
Source: Obes Sci Pract. 2025 Mar 22;11(2):e70048. doi: 10.1002/osp4.70048 (PMC11929140; doi:10.1002/osp4.70048)
Supplement: Supplementary file 1 — Supporting Information S1 [file OSP4-11-e70048-s001.docx]

**Acceptability and feasibility of an acceptance-based guided self-help programme for weight loss maintenance in adults who have previously completed a behavioural weight loss programme: the SWiM feasibility study: SUPPLEMENTARY MATERIALS**

Table S1. Characteristics of the interviewed intervention and control participants.

| Characteristic | Number and percentage of intervention participants (unless otherwise stated) | | Number and percentage of control participants (unless otherwise stated) | |
| --- | --- | --- | --- | --- |
| Age (mean [min – max]) | 54 (24 – 73) | | 51 (35 – 66) | |
| Female sex | 15/18 (83%) | | 8/10 (80%) | |
| White ethnicity | 16/17 (94%)  Not reported = n=1/18 | | 8/8 (100%)  Not reported = 2/10 | |
| Occupation level | Employee (full-time)  Employee (part-time)  Self-employed  Voluntary work Permanently sick/disabled  Wholly retired  Other  Not reported | 7/18  2/18  0/18  1/18  1/18  4/18  2/18  1/18 | Employee (full-time)  Employee (part-time)  Self-employed  Voluntary work Permanently sick/disabled  Wholly retired  Other  Not reported | 3/18  1/18  2/18  0/18  1/18  2/18  0/18  1/18 |


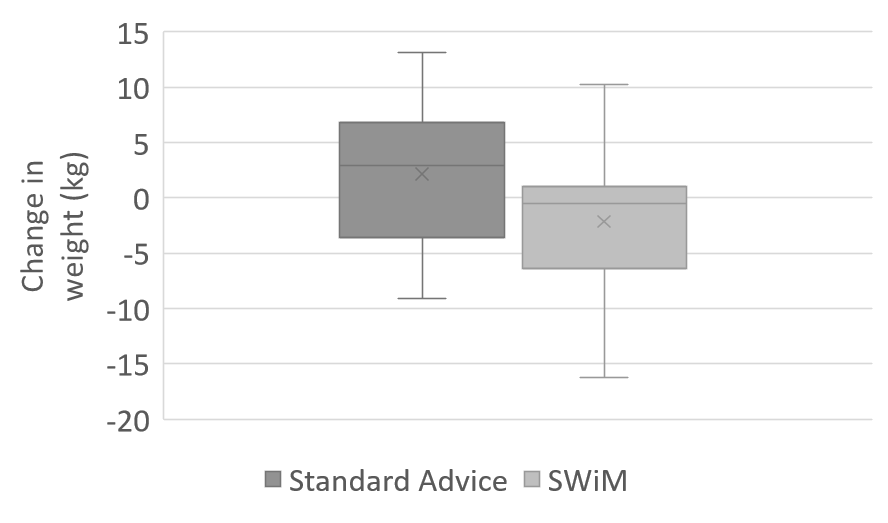


Figure S1. Box plots for change in weight from baseline to 6 months in the two study groups (intervention = SWiM, control = standard advice).


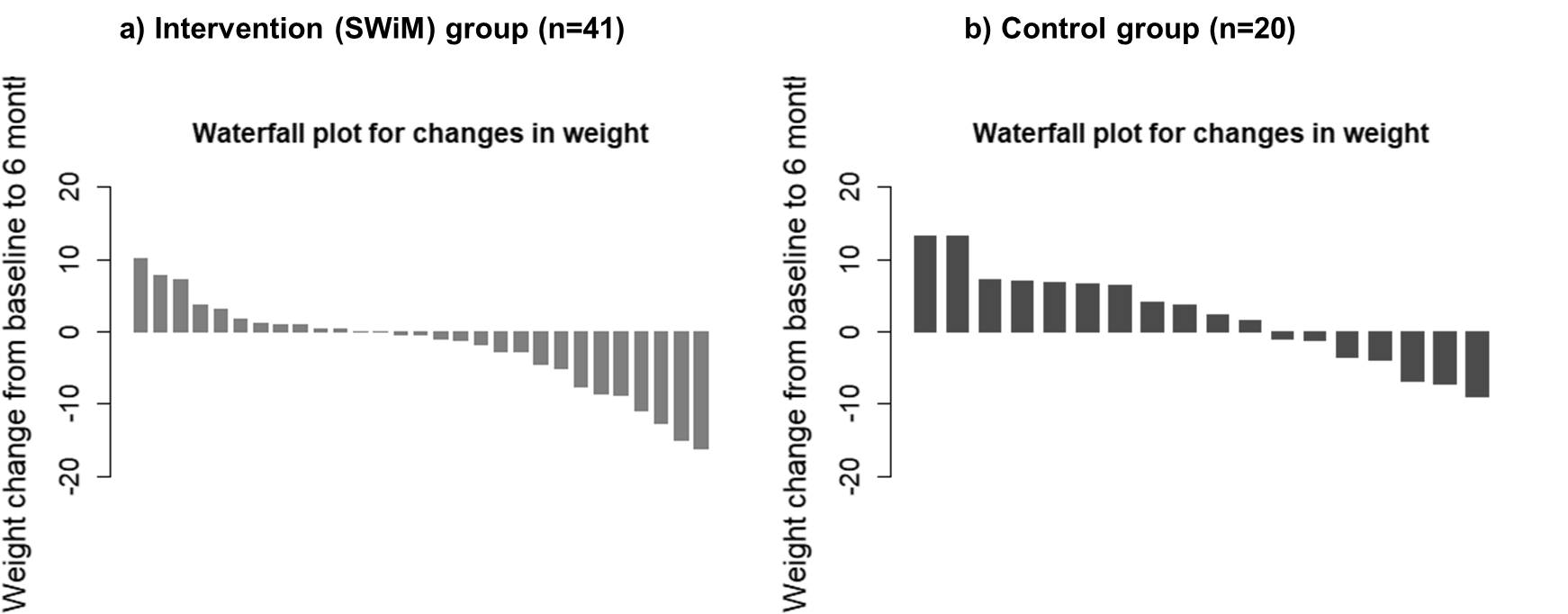


Figure S2. Waterfall charts showing weight change from baseline to 6 months in a) the intervention group (i.e. the SWiM group) and b) the control group.


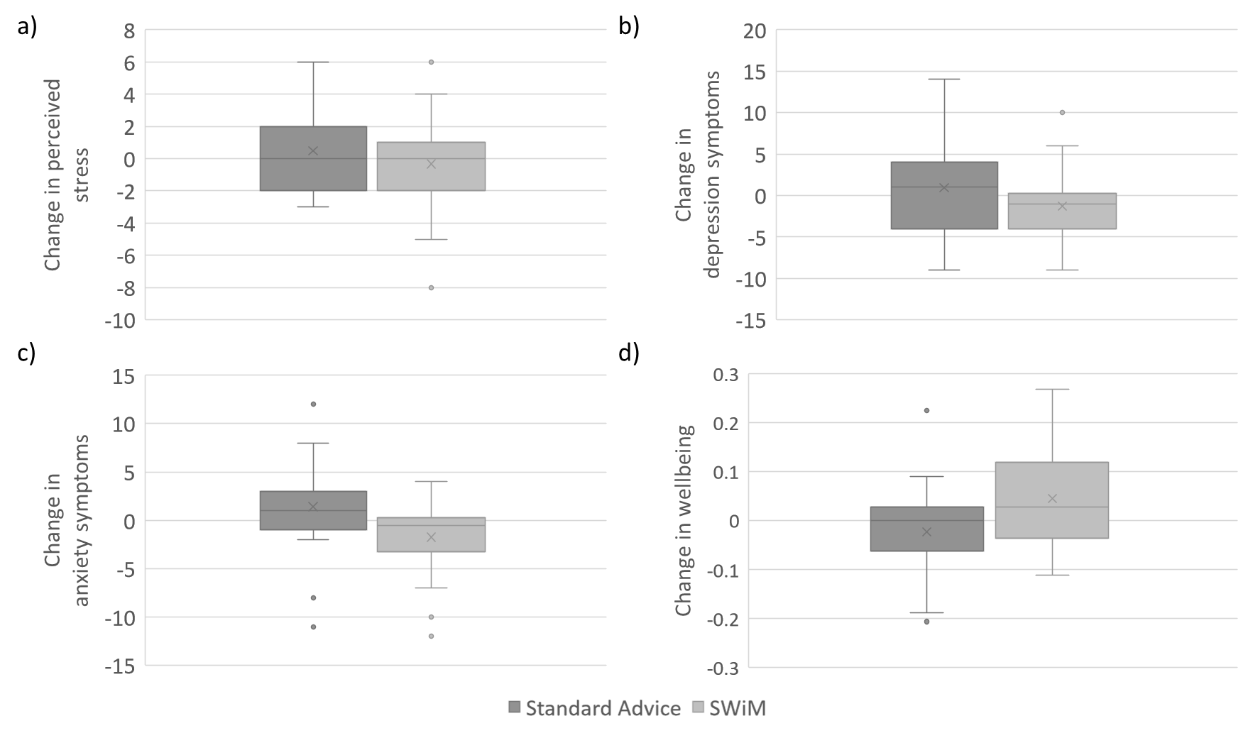


Figure S3. Boxplots showing change from baseline to 6 months in a) perceived stress (Perceived Stress Scale, PSS-4), b) depressive symptoms (Patient Health Questionnaire, PHQ-8), c) anxiety symptoms (Generalised Anxiety Disorder questionnaire, GAD-7), and d) wellbeing (ICEpop CAPability measure for Adults, ICECAP) in the two study groups (Intervention = SWiM, Control = standard advice).


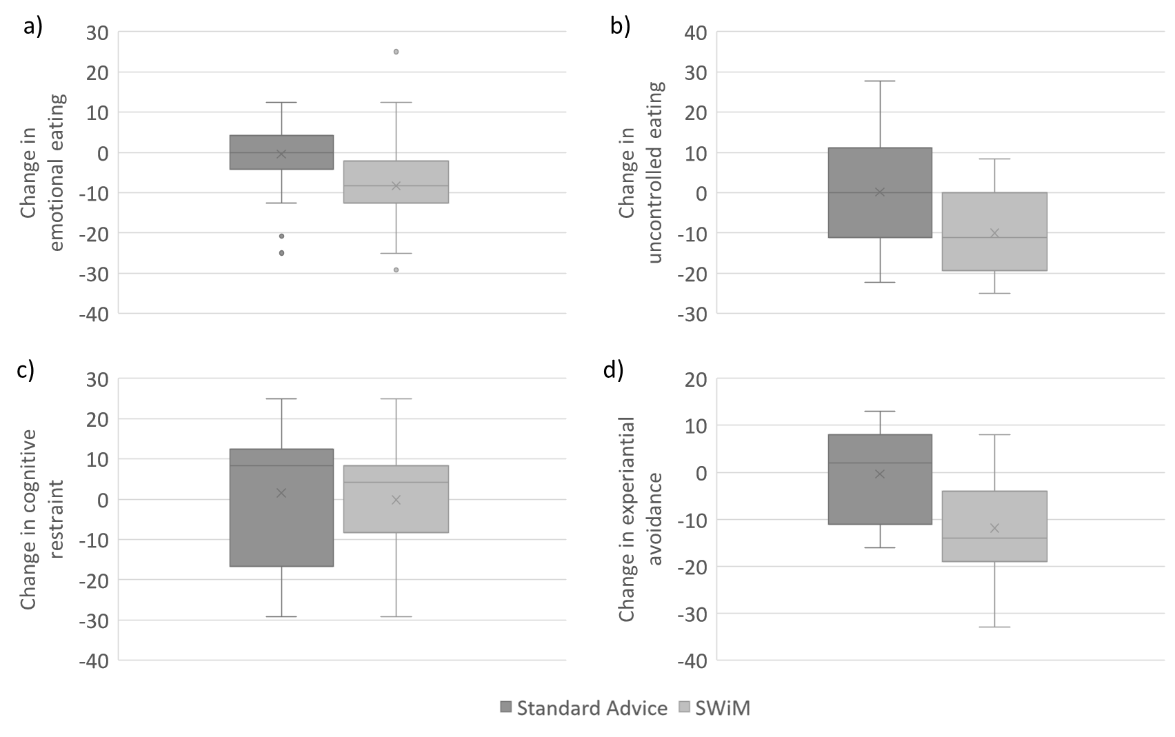


Figure S4. Boxplots showing change from baseline to 6 months in a) emotional eating (Three-Factor Eating Questionnaire [TFEQ]), b)uncontrolled eating (TFEQ), c) cognitive restraint of food intake (TFEQ), and d) experiential avoidance/psychological flexibility (Acceptance and Action Questionnaire for Weight-Related Difficulties-Revised (AAQW-R) in the two study groups (Intervention = SWiM, Control = standard advice).

**Habit strength questionnaire**

1. **Eating high-calorie snacks when I feel stressed is something I do automatically**

*1 (Strongly disagree)*

*2*

*3*

*4 (Neither agree nor disgaree)*

*5*

*6*

*7 (Strongly agree)*

1. **Eating high-calorie snacks when I feel stressed is something I do without thinking**

*1 (Strongly disagree)*

*2*

*3*

*4 (Neither agree nor disgaree)*

*5*

*6*

*7 (Strongly agree)*

1. **Eating high-calorie snacks when I feel tired is something I do automatically**

*1 (Strongly disagree)*

*2*

*3*

*4 (Neither agree nor disgaree)*

*5*

*6*

*7 (Strongly agree)*

1. **Eating high-calorie snacks when I feel tired is something I do without thinking**

*1 (Strongly disagree)*

*2*

*3*

*4 (Neither agree nor disagree)*

*5*

*6*

*7 (Strongly agree)*

1. **Eating high-calorie snacks when I feel ‘down’ is something I do automatically**

*1 (Strongly disagree)*

*2*

*3*

*4 (Neither agree nor disagree)*

*5*

*6*

*7 (Strongly agree)*

1. **Eating high-calorie snacks when I feel ‘down’ is something I do without thinking**

*1 (Strongly disagree)*

*2*

*3*

*4 (Neither agree nor disagree)*

*5*

*6*

*7 (Strongly agree)*

1. **Eating high-calorie snacks when I feel anxious is something I do automatically**

*1 (Strongly disagree)*

*2*

*3*

*4 (Neither agree nor disagree)*

*5*

*6*

*7 (Strongly agree)*

1. **Eating high-calorie snacks when I feel anxious is something I do without thinking**

*1 (Strongly disagree)*

*2*

*3*

*4 (Neither agree nor disagree)*

*5*

*6*

*7 (Strongly agree)*

1. **Eating high-calorie snacks when I feel happy is something I do automatically**

*1 (Strongly disagree)*

*2*

*3*

*4 (Neither agree nor digagree)*

*5*

*6*

*7 (Strongly agree)*

1. **Eating high-calorie snacks when I feel happy is something I do without thinking**

*1 (Strongly disagree)*

*2*

*3*

*4 (Neither agree nor disagree)*

*5*

*6*

*7 (Strongly agree)*
